# The influence of academic examinations on energy and nutrient intake in male university students

**DOI:** 10.1186/s12937-015-0088-y

**Published:** 2015-09-25

**Authors:** Margo E. Barker, Richard J. Blain, Jean M. Russell

**Affiliations:** 1 Department of Oncology, Human Nutrition Unit, Medical School, University of Sheffield, Sheffield, S102RX UK; 2 Corporate Information and Computing Service, University of Sheffield, Sheffield, UK

**Keywords:** United Kingdom, Diet, Nutrition, Stress, Hypophagia, Hyperphagia

## Abstract

**Background:**

Taking examinations is central to student experience at University and may cause psychological stress. Although stress is recognised to impact on food intake, the effects of undertaking examinations on students’ dietary intake have not been well characterised. The purpose of this study was to assess how students’ energy and nutrient intake may alter during examination periods.

**Methods:**

The study design was a within-subject comparison of students’ energy and nutrient intake during an examination period contrasted with that outside an examination period (baseline). A total of 20 male students from the University of Sheffield completed an automated photographic 4-d dietary record alongside four 24-h recalls in each time period. Daily energy and nutrient intake was estimated for each student by time period and change in energy and nutrient intake calculated. Intakes at baseline were compared to UK dietary recommendations. Cluster analysis categorised students according to their change in energy intake between baseline and the examination period. Non-parametric statistical tests identified differences by cluster.

**Results:**

Baseline intakes did not meet recommendations for energy, non-milk extrinsic sugars, non-starch polysaccharide and sodium. Three defined clusters of students were identified: Cluster D who decreased daily energy intake by 12.06 MJ (*n* = 5), Cluster S who had similar energy intakes (*n* = 13) and Cluster I who substantially increased energy intake by 6.37 MJ (*n* = 2) between baseline and examination period. There were statistically significant differences (all *p* < 0.05) in change in intake of protein, carbohydrate, calcium and sodium between clusters. Cluster D recorded greater energy, carbohydrate and protein intakes than Cluster I at baseline.

**Conclusions:**

The majority of students were dietary resilient. Students who demonstrated hypophagia in the examination period had a high energy and nutrient intake at baseline, conversely those who showed hyperphagia had a low energy and nutrient intake. These patterns require confirmation in studies including women, but if confirmed, there is need to address some students’ poor food choice especially during examinations.

## Introduction

Taking academic examinations is a core part of student experience at university. University Student Services [1] advise eating well (regular meals and eating breakfast), but the dietary habits of students during examination periods have not been closely scrutinized.

The impact of examinations on eating behaviours of students has been studied from a stress perspective. It is recognized that undergoing examinations is psychologically stressful; heightened anxiety and emotional distress have been documented in students during examination periods [2, 3], as well as surges in stress hormones such as cortisol and adrenocorticotropic hormone [4–6]. A study of female students reported that that disordered eating traits (dietary restraint, bulimia, oral control) were more prevalent during examination periods [7], while seemingly contrasting effects, namely, an increased desire to eat and greater eating frequency were observed in a study of both male and female students taking examinations [8]. Examination stress may impact on alcohol consumption patterns, for example students with good social support reported lower alcohol consumption in examination periods compared to a control time period, while conversely students lacking social support reported greater alcohol consumption [2]. Similarly, Pollard et al. noted that students classified as having low social support had increased energy and fat intake during examination periods [9]. This study also concluded that the overall effect of examination stress on energy and nutrient intake was minor, with no change in number of eating occasions, nor energy, fat, saturated fat, starch or sugars intake [9]. However, diet was assessed over a single 24-h period, which may be too short to delineate changes in eating patterns and provide reliable estimates of energy and nutrient intake.

Possible alterations in eating behaviour in student populations during examination periods have been underpinned by evidence from community studies, which have reported a preference for hedonic, snack-type, energy-dense, high-fat foods in persons experiencing chronic psychological stress relative to non-stressed persons [10, 11]. It has been proposed that the act of eating may aid emotional regulation, and consumption of “comfort food” high in fat, sugar and carbohydrate, may attenuate negative psychological states [12, 13]. In student populations, perceived psychological stress (not specific to academic or examination stress) has been documented to associate variously with greater consumption of fast, sweet and snack foods, low fruit and vegetable consumption and binge drinking [14–18]. There are indications that these effects on food consumption may be stronger in women [14, 16, 18], although some studies did not explore the influence of gender [15, 17].

It is generally accepted that stress can have a bi-directional effect on appetite resulting in both hyperphagia and hypophagia [14, 15, 17, 19], and there is evidence that appetite response is modulated by gender, restrained eating, and baseline Body Mass Index (BMI) [10, 19, 20]. People who have been categorised as “emotional eaters” (eating to counteract negative emotions such as fear, sadness and anxiety) also record a predilection for high-fat and/or high-sugar “comfort” food in stressful situations [21–23].

The precise effects of how taking examinations may impact on students’ energy and nutrient intake are unclear. Research into such effects has been limited because dietary assessment methods that assess contemporaneous dietary intake such as food diaries are time-consuming, rely on self-report and have the potential to interfere with eating routines. Studies that involve high workload are unfeasible in the immediate lead-up to examinations when time pressures are great. Furthermore, less demanding dietary assessment methods, such as a single 24-h dietary recall may lead to misclassification of energy and nutrient intake because of within-subject variation in energy and nutrient intake. This exploratory study seeks to ascertain how students’ energy and nutrient intake may be altered during examination periods using an automated photographic dietary assessment method over a four-day time period.

## Methods

### Study design

The study was designed as a within-subject comparison of students’ dietary intake during an examination period (defined as three calendar weeks prior to an examination) and outside an examination period. The non-examination period included both teaching and vacation times. Ethical approval for the study was obtained through the School of Medicine’s ethical review procedure at the University of Sheffield.

### Subjects

Subjects were recruited via email using University of Sheffield mailing lists. The email described the recruitment criteria, basis of the study and contact details of the researchers. Subjects were eligible if they were male university students undertaking an examination within the timeframe of the study (May to August 2014). Male, as opposed to female, students were chosen, as the study would have had insufficient statistical power to adjust for fluctuations in energy intake across the menstrual cycle [24]. International students were excluded in order to reduce variance in types of foods consumed and facilitate coding of food intake for nutrient analysis. Students enrolled on nutrition programmes were excluded as they have been shown to have non-standard eating behaviours [25, 26]. Interested subjects were supplied with an information sheet detailing the study and participant requirements. In total, forty-three individuals expressed interest, twenty-three did not take part, as some did not follow through initial interest (*n* = 6) and some were ineligible (*n* = 17). The final sample size was 20 subjects. All subjects gave written informed consent.

The following subject information was collected: contact details, age, ethnicity, course, level and year of study and examination dates. Participants were also asked to state whether or not they lived in catered student halls.

### Dietary assessment

Dietary intake was assessed using combined automated photographic records with multiple pass 24-h recalls. The use of wearable cameras for dietary assessment has been previously validated with athletes and students [27, 28] and has been shown to be superior to 24-h recall methods when measured against energy expenditure [29]. For this study the Autographer camera was used to record food intake. The Autographer is a lightweight, wearable, digital camera (58 g, 37.4 mm × 95.5 mm × 22.93 mm) with a 136° wide-angle lens and having an internal memory of 8GB. The camera hangs on a lanyard around the neck resting above the chest bone. Images are captured automatically every 15–30 s or in reaction to movement, or change in light. The user interface displays battery life, number of images taken, and percentage of memory remaining. The camera is provided with a software package, which allows uploading of images on a computer via USB connectivity. Images can be viewed by date of capture.

All participants attended a briefing session where the camera was introduced and its use explained. Subjects were shown how to use the camera, with emphasis placed on personal privacy. For example, participants were told to remove the camera when using the bathroom, changing clothes, attending the gym or in any scenario they deemed private. Participants wore the camera for eight days in total: four days within the examination period and four days in the non-examination period. Each four-day period comprised three weekdays and one weekend day. Each participant wore the camera from early morning to bedtime. Participants were asked to charge the camera overnight. The following day each participant met with the lead researcher in a University venue to conduct a 24-h recall.

The multiple pass 24-h dietary recall was performed in accordance with the method of Gibson [30] adapted to include the photographic images. Firstly, the photographic images were uploaded to the researcher’s laptop; this process took 10–15 min. During this time the first two passes of the 24-h recall were conducted. For the third pass of the recall the researcher and participant together reviewed the previous day’s food images on the laptop. This visual review served as a memory cue, allowing the participant to remember further details of reported items, including: portion size, brand names, condiments and ingredients in meals. Viewing the photographic images allowed the identification of omissions from the initial two passes of the recall. It also provided opportunities to probe and seek clarification on any vague or unfamiliar details. The fourth pass comprised a review of all reported food and drink items.

Upon completion of the 24-h recall, the camera was returned to the participant to wear for the remainder of that day. This was the procedure employed throughout the duration of the study. During the examination period, three students notified the researcher that they could not meet due to revision commitments. In these cases the first two passes of recalls were carried out by telephone and email. The researcher then downloaded and reviewed all images. Discrepancies between the previous accounts and the automated photographic record were clarified with the student.

### Data analysis

Average daily nutrient intake was calculated from the four-day recalls using NetWisp, Version 4.0 (Tinuviel Software, Warrington, UK) on a University networked computer. NetWisp uses the most up-to-date McCance and Widdowson nutrient databank to calculate nutrient intakes. Portion sizes were estimated using the downloaded photographic images in conjunction with a photographic food portion size book [31]. Mean daily energy and nutrient intake was generated for each subject.

Descriptive statistics of nutrient intake for the sample were generated at the two time points. Median nutrient intakes outside of the examination period were compared to UK Dietary Reference Values (DRVs) [32, 33] using a non-parametric Wilcoxon Signed Rank Test. The change in energy intake between time points was calculated. Hierarchical cluster analysis, using Wards Agglomeration Method applying squared Euclidean distance, was performed on this variable (change in energy intake) to generate three clusters of subjects. Kruskal- Wallis tests were then conducted on change in intakes of protein, fat, saturated fat, carbohydrate, non-milk extrinsic sugars (NMES), alcohol, non-starch polysaccharide (NSP), calcium and sodium in relation to cluster membership. The SPSS software package V22.0 (SPSS Statistics, International Business Machines, New York) was used for statistical analysis.

## Results

### Subject characteristics

Of the twenty participants who took part in the study, all were male full-time students living in non-catered accommodation. Three students were taking taught postgraduate programmes; the remaining 17 students were at undergraduate level. The median age of the sample was 20.0 years, with an age range of 18–25 years. The majority of students were of White ethnicity (*n* = 16), with the remainder being of Mixed Race (*n* = 2) and Asian (*n* = 2) ethnicities. The median number of examinations taken was 3.0.

### Energy and nutrient intake

Table 1 provides median intakes of energy and nutrients during the non-examination time period in comparison to UK Dietary Reference Values [32, 33]. Energy intake was significantly lower (*p* = 0.021) than the Estimated Average Requirement (EAR). Intakes of protein and calcium were significantly greater (*p* < 0.001 and *p* = 0.033, respectively) than Reference Nutrient Intake (RNI) values. Fat energy and saturated fat energy exceeded, but were not significantly different from DRV population average targets of 35 % energy (*p* = 0.279) and 10 % energy (*p* = 0.117), respectively. The contribution of non-milk extrinsic sugars (NMES) to total energy intake was significantly above (*p* < 0.001) the recommended value. NSP intake was significantly lower (*p* = 0.002) than the DRV recommendation of 18 g/d. The median intake of alcohol energy was lower than the DRV recommendation, however this difference was not statistically significant (*p* = 0.784), and there was a wide range of values.

**Table 1:** Energy and nutrient intake of students outside of examination periods (baseline) compared to UK Dietary Reference Values^1^

	Median intake (Range)	DRV	Difference from DRV	*P* value
Total energy (MJ/d)	10.7 (5.9 – 13.5)	11.6^1^	- 0.89	0.021
Protein (g/d)	93.9 (49.7 – 177.2)	55.5	+38.4	0.000
Total fat (% total energy)	36.1 (22.6 – 46.9)	33	+3.1	0.279
Saturated fat (% total energy)	11.4 (5.5 – 19.8)	10	+1.4	0.117
Carbohydrate (% total energy)	45.7 (32.9 – 53.1)	50	- 4.3	0.001
NMES (% total energy)	19.0 (9.1 – 54.9)	10	+9.0	0.000
Alcohol (% total energy)	2.7 (0.0 – 27.9)	5	- 2.3	0.910
NSP (g/d)	13.5 (3.7 – 21.6)	18	- 4.5	0.002
Calcium (mg/d)	861 (389 – 1380)	700	+161	0.033
Sodium (mg/d)	3115 (1494 – 4921)	2500	+613	0.006

*NMES* non-milk extrinsic sugars, *NSP* non-starch polysaccharide

^1^The Estimated Average Requirement value has been used for energy. Reference Nutrient Intake values have been used for protein and calcium. Population average intakes have been used for fat, saturated fat, carbohydrate, NMES, NSP and alcohol

Cluster analysis identified three defined clusters of subjects according to change in energy intake in the examination period relative to the non-examination period: Cluster D who decreased energy intake (*n* = 5), Cluster S who had similar energy intakes (*n* = 13) and Cluster I who substantially increased energy intake (*n* = 2). The median number of examinations taken according to Cluster membership was: 3.0 for Cluster D, 3.0 for Cluster S and 3.5 for Cluster I.

There were significant differences in nutrient intake between clusters at baseline. Table 2 provides a description of nutrient intake by cluster. Cluster D recorded greater energy, carbohydrate and protein intakes than Cluster I. Intakes of fat, saturated fat, NMES, alcohol, NSP, calcium and sodium were similar in all three clusters at baseline. The two students in Cluster I recorded daily energy intakes of 6.343 MJ kJ (1503 kcal) and 6.41 MJ 0 kJ (1519 kcal) in the non-examination period.

**Table 2:** Baseline daily energy and nutrient intake by cluster membership; median values and 95 % confidence intervals in parentheses

Nutrient	Cluster D	Cluster S	Cluster I	*P* value
	Energy decrease (*n* = 5)	Similar energy intake (*n* = 13)	Energy increase (*n* = 2)	
Energy (MJ/d)	12.06 (10.31, 13.66)	10.41 (6.01,11.66)	6.38 (6.34,6.41)	0.014
Protein (g/d)	96.9 (95.7,132.1)	88.9 (56.6,129.7)	56.4 (49.7,63.0)	0.030
Fat (g/d)	85.0 (75.0,104.3)	83.1 (66.3,127.8)	69.5 (61.5,77.4)	0.421
Saturated fat (g/d)	35.6 (18.6,37.2)	30.2 (12.8,40.8)	22.5 (17.7,27.3)	0.555
Carbohydrate (g/d)	293.3 (253.7,410.8)	256.4 (164.0,304.9)	156.0 (126.4,185.6)	0.022
NMES (g/d)	117.8 (63.2,229.5)	95.3 (37.6,134.7)	66.1 (51.4,80.8)	0.265
Alcohol (g/d)	20.3 (0.0,125.2)	1.5 (0.0,27.4)	10.9 (0.0,21.7)	0.551
NSP (g/d)	14.3 (5.7,21.6)	13.9 (10.4,16.7)	10.8 (9.8,11.7)	0.476
Calcium (mg/day)	898 (388,1380)	902 (393,1143)	48.0 (415,545)	0.179
Sodium (mg/d)	3418 (1973,4758)	2995 (2003,4318)	2045 (1494,2596)	0.160

*NMES* non-milk extrinsic sugars, *NSP* non-starch polysaccharide

Table 3 details changes in nutrient intake between baseline and examination period according to cluster membership. There were statistically significant differences in change in intake of protein (*p* = 0.034), carbohydrate (*p* = 0.012), calcium (*p* = 0.019) and sodium (*p* = 0.031) between clusters. There was a substantial fall in intake of these nutrients for Cluster D while Cluster I recorded substantial increases. Changes in intake of total fat and NMES were of borderline statistical significance (*p* = 0.059 and *p* = 0.054, respectively) and followed the same pattern of change by cluster membership as for other macronutrients. In contrast there was only marginal change in intake of all nutrients for Cluster S students. Changes in alcohol and NSP intakes were not significantly different between the three clusters.

**Table 3:** Change in nutrient intakes by cluster membership; median values and 95 % confidence intervals in parentheses

Nutrient	Cluster D	Cluster S	Cluster I	*P* value
	Energy decrease (*n* = 5)	Similar energy intake (*n* = 13)	Energy increase (*n* = 2)	
Energy (MJ/d)	−2.59 (−6.38,-2.01)	+0.22 (−5.53.,2.25)	+6.00 (5.85, 6.15)	n/a*
Protein (g/d)	−28.0 (−58.4, 2.9)	−3.8 (−16.3,20.4)	+12.6 (5.1, 20.0)	0.034
Fat (g/d)	−22.2 (−31.1,22.3)	+13.5 (−31.9,33.0)	+53.4 (39.2,67.6)	0.059
Saturated fat (g/d)	−4.6 (−10.8,3.5)	+12.2 (−16.3,24.6)	+29.9 (25.2, 34.6)	0.066
Carbohydrate (g/d)	−33.0 (−209.4,48.1)	+11.6 (−35.2,55.5)	+207.7 (138.1, 277.3)	0.012
NMES (g/d)	−26.7 (−91.2,73.8)	3.4 (−34.0,12.4)	+166.8 (141.5, 192.2)	0.054
Alcohol (g/d)	−18.7 (−105.6,27.4)	4.7 (−19.6,15.0)	+17.7 (−1.1, 36.6)	0.426
NSP (g/d)	−1.6 (−12.9,6.0)	−1.2 (−4.8, 1.9)	−0.350 (−1.4,0.70)	0.784
Calcium (mg/day)	−172 (−821,-48)	144 (−200,231)	147 (80,215)	0.019
Sodium (mg/d)	−758 (−2510,626)	26 (−470,1418)	1134 (317, 1951)	0.031

*NMES* non-milk extrinsic sugars, *NSP* non-starch polysaccharide

*Data clustered on this variable

## Discussion

In this exploratory study there was no evidence of a change in appetite during the examination period for the majority of students: the largest cluster of students (Cluster S), comprising 65 % of our sample, appeared to be dietary resilient. These students reported small shifts in intakes of protein, fat, carbohydrate, sugars and alcohol during the examination period, but the combined effect did not impact on total energy intake. This inertia in energy intake concurs with the conclusion of a directly comparable study of students’ energy and macronutrient intake in relation to examination stress [9]. Our identification of a group of students who seem to be dietary resilient to examination stress at a total daily consumption level, is somewhat at odds with general understandings as to the effects of stress on food intake and appetite, which suggest that stress is associated with both increased and decreased eating [19, 34]. Although some observational studies of self-perceived appetite in response to stress have identified subjects who report eating the same, this subject group was in the minority, comprising approximately 10 to 20 % of the sample [14, 17, 35]. The fact that the current study had an all-male sample may account for the discrepancy, since men may be less prone to “emotional eating” [10], although the fact that consumption was assessed precisely, as opposed to subjective recall of appetite, may be a more compelling explanation.

The dietary inertia of Cluster S students also extended beyond the main nutrient energy sources; intakes of NSP, saturated fat, NMES, calcium and sodium were similar in the examination period compared to baseline. The lack of change in intake of these nutrients indicates that dietary patterns were largely unchanged, with no shift towards snack-type food of low nutrient density that is high in sugars, salt and fat, although baseline intakes of NMES and sodium were already high and NSP was low. This maintenance of the dietary status quo contrasts with the conclusions of community-based studies that have examined self-reported consumption of specific foods in relation to chronic self-perceived stress [15, 18, 36–39]; these have reported that stress manifests in greater consumption of palatable, fatty and sweet foods, and conversely lesser intake of salads, fruit and vegetables. Also, in keeping with the behaviour of Cluster S students, laboratory studies of experimentally-induced stress report no change in food choice at a meal level [21]. Other laboratory studies report increased consumption of snack, sweet and fatty foods following stress [36, 37], especially amongst subjects characterised as “emotional eaters” [21, 40]. Notably men’s food choice seems to be less susceptible to the effects of stress than women’s in both observational and experimental situations [18, 41].

The substantial changes in energy and nutrient intake observed in Cluster D and Cluster I students, who decreased and increased intake, respectively, concur with the literature on stress and hypophagia and hyperphagia [19, 21]. Interestingly, the direction of change in energy intake was related to baseline energy intake. Cluster D students, who decreased energy intake, had substantially greater energy and carbohydrate intake at baseline than other clusters. These effects are in accord with a study of school children undertaking examinations, which documented that those who had a high energy intake on the control day decreased their intake on the examination day [42]. Conversely Cluster I students increased energy intake by almost 100 % in the examination period and had extremely low intakes of energy, protein and carbohydrate at baseline. The behaviour of Cluster I students in relation to their baseline energy intake fits with known interactions between dieting status, dietary restraint and stress-induced eating [10, 36, 39, 43], although not all studies have observed that dietary restraint is associated with a strong stress and hyperphagia response [35, 37]. However, without psychometric measures of eating behaviour, covering dietary restraint, disinhibition and emotional eating, interpretation of the interaction between baseline energy and hyperphagia can only be speculative.

Of note, students in cluster D who decreased energy intake achieved this largely by decreasing intake of protein, fat and alcohol, while the decrease in intake of carbohydrate and NMES was less. This observation infers that hypophagia does not extend to intake of carbohydrate-rich foods. It is difficult to contextualise this finding, as studies that have described stress hypophagia do not detail changes in intake of major nutrients or food groups [14, 17]. Cluster D was small in size and this effect requires corroboration in larger studies.

The substantial alterations in energy and nutrient intake observed in two clusters must be seen against the backdrop of students’ poor diet choice at baseline. Although the group as whole recorded energy intakes close to recommendations, their intakes of NMES and sodium were greater than recommended while NSP was low. Studies of students report low fruit and vegetable intake both in the UK [16] and internationally [44].

The study has a number of important limitations. Due to the study being carried out in a short time window the sample size is small and inferences we can draw are therefore limited. The small number of students in Cluster I makes drawing conclusions about the prevalence of examination induced hyperphagia particularly tenuous. The dietary methodology necessitated intensive interaction with students and this acted to constrain the sample size. Ideally dietary monitoring would have been conducted in the calendar week immediately preceding an examination, but resource constraints dictated a wider window. Furthermore we lacked measurements of BMI and body weight change, which would have provided nuance to interpretation of the differences in change in energy intake across clusters. Equally, a questionnaire measure of stress would have given quantitative confirmation of the impact of the examination, while psychometric data on eating behaviour traits would have added to interpretation of the seemingly various effects on appetite. These data clearly require corroboration in a larger study, which includes women, for whom emotional eating may be more prevalent. The interaction between food choice during examinations, and caffeine use, smoking habits and exercise patterns should be explored.

## Conclusions

Despite these limitations, this exploratory study has elucidated how the dietary behaviour of students varies under academic stress. As such it is one of the few studies that measures diet at a total level during an examination time period. We conclude that there are different dietary responses in relation to taking examinations: a major group of students seem dietary resilient, a very small minority of students show hyperphagia and a larger minority of students display hypophagia. It seems as if a minority of student make risky food choices during examination periods. A better understanding of these effects could lead to a more focussed dietary advice for students taking examinations, including the provision of nuanced advice for both hypophagia and hyperphagia.

### Availability of supporting data

The data set supporting the results of this article is available in the Figshare repository, http://figshare.com/articles/Examination_Students_Data/1332430.

## Abbreviations

NMES: Non -milk extrinsic sugars
DRV: Dietary reference values
NSP: Non-starch polysaccharides
EAR: Estimated average requirement
RNI: Reference nutrient intake
